# Elevated body mass index and clinical outcomes after surgical stabilization for shoulder instability: A systematic review

**DOI:** 10.1016/j.jor.2026.02.052

**Published:** 2026-02-16

**Authors:** Bryce C. Johnson, Erryk S. Katayama, Benjamin L. Brej, Kavya B. Ajjarapu, Julie Y. Bishop, Gregory L. Cvetanovich, Ryan C. Rauck

**Affiliations:** a Department of Orthopaedics, The Ohio State University College of Medicine, Columbus, OH, USA; b Investigation Performed at the Ohio State University College of Medicine, Columbus, OH, USA

**Keywords:** Obesity, Body mass index, Shoulder, Instability, Surgery, Stabilization, Outcomes

## Abstract

**Background:**

Obesity is understood to be a potential risk factor that negatively impacts outcomes across various orthopaedic procedures including weightbearing joint arthroplasty, and even in non-weightbearing joints, such as total shoulder arthroplasty and rotator cuff repair. However, there is limited understanding of the role obesity plays in the incidence and outcomes of shoulder instability.

**Hypothesis/purpose:**

The purpose of this study is to characterize the relationship between body mass index (BMI) and shoulder instability outcomes following operative stabilization.

**Study design:**

This systematic review was conducted using the MEDLINE, Embase, and Cochrane databases to identify studies investigating shoulder instability surgery. Studies were included that involved patients undergoing shoulder stabilization surgery and investigated BMI or obesity as a variable. Exclusion criteria included non-stabilizing shoulder procedures, joints other than the shoulder, case reports or case series with less than 5 cases, and secondary literature.

**Methods:**

Two authors independently reviewed all relevant articles using the inclusion and exclusion criteria mentioned above and any discrepancies were addressed by a third independent reviewer. Data extracted from each study included patient demographics, procedure type and operative findings, and functional outcome measures, including patient-reported outcomes (PROs).

**Results:**

A total of 936 patients were identified across the 7 included studies and the mean BMI for this cohort was 29.74 kg/m^2^. Increased BMI did not emerge as an independent predictor of surgical failure or recurrent instability following operative stabilization. However, several meaningful trends did reach statistical significance in the elevated BMI group. We found that individuals with increased BMI were more likely to need surgical intervention, demonstrated higher levels of surgical complexity, and decreased subjective improvement thresholds determined by MCID. They also showed higher rates of specific injury patterns including partial rotator cuff tears, Bankart lesions, and bone and cartilage lesions (BCLs). Patients with elevated BMI were also less likely to return to sport following certain procedures.

**Conclusion:**

Our findings suggest that while obesity may not directly drive surgical failure, it has a significant role in determining technical complexity and the rehabilitation experience for the patient.

## Highlights

• Elevated BMI was not an independent predictor of instability recurrence and inferior outcomes following surgery.
• Obesity was associated with increased surgical complexity, specific injury patterns, and decreased return to sport following surgery.
• Obesity influences injury severety and postoperative functional perception.

## 1 Introduction

Obesity rates are alarmingly high in the United States and continue to rise with a current national prevalence of over 40% and projections to increase to over 50% by 2030.^1^^,^^2^ Obesity has been broadly associated with worse outcomes across orthopedic procedures, particularly weightbearing lower extremity surgeries such as total hip and total knee arthroplasty, including longer operative times, higher complication rates, and greater healthcare costs.^3^^,^^4^ However, data on non-weightbearing joints like the shoulder is more limited. Recent studies examining body mass index (BMI) in shoulder arthroplasty and rotator cuff repair suggest that obesity may also negatively affect postoperative outcomes, but there remains a paucity of research investigating similar trends in shoulder instability.^5^, ^6^, ^7^.

The glenohumeral joint is the least stable and most frequently dislocated major joint in the body, with estimated instability prevalence rates of 62 in 100,000 people.^8^^,^^9^ Instability is especially common in active individuals, such as contact or overhead sport athletes, who place increased stress on the joint.^10^, ^11^, ^12^ Injury patterns may involve soft tissue structures, including the glenohumeral capsule and labrum, typically addressed with Bankart repair, or more significant bony involvement with glenoid bone loss requiring bone block transfer procedures such as the Latarjet. Increased BMI may alter shoulder mechanics and place greater mechanical stress across the glenohumeral joint. However, recent studies suggest that obesity may not be as detrimental to outcomes in shoulder instability as previously thought.^13^

The aim of this study is to better characterize the association between obesity and both the incidence and outcomes of shoulder instability following operative stabilization. Herein, we conducted a systematic review to analyze existing literature and better understand how BMI impacts clinical and patient-reported outcomes after shoulder stabilization surgery.

## 2 Methods

A systematic review was conducted using the MEDLINE, Embase, and Cochrane databases to identify studies examining the relationship between BMI and shoulder instability surgery. The following search strategy was used: (“body mass index” OR “BMI” OR “index, body mass”) AND (“shoulder” OR “glenohumeral”) AND (instab∗ OR dislocat∗ OR subluxat∗) AND (recur∗ OR revis∗ OR fail∗) AND (surg∗ OR operat∗ OR stabil∗ OR Latarjet OR Bankart OR labrum OR arthroscop∗ OR remplissage OR capsulorrhaphy). The search was completed on October 27, 2024. The review adhered to PRISMA (Preferred Reporting Items for Systematic Reviews and Meta-Analyses) guidelines. Using the PICO framework, we investigated: (Population) patients with shoulder instability, (Intervention) surgical stabilization, (Comparison) varying BMI levels, and (Outcome) rates of recurrent instability or patient-reported outcomes following surgery.

**Fig. 1:**
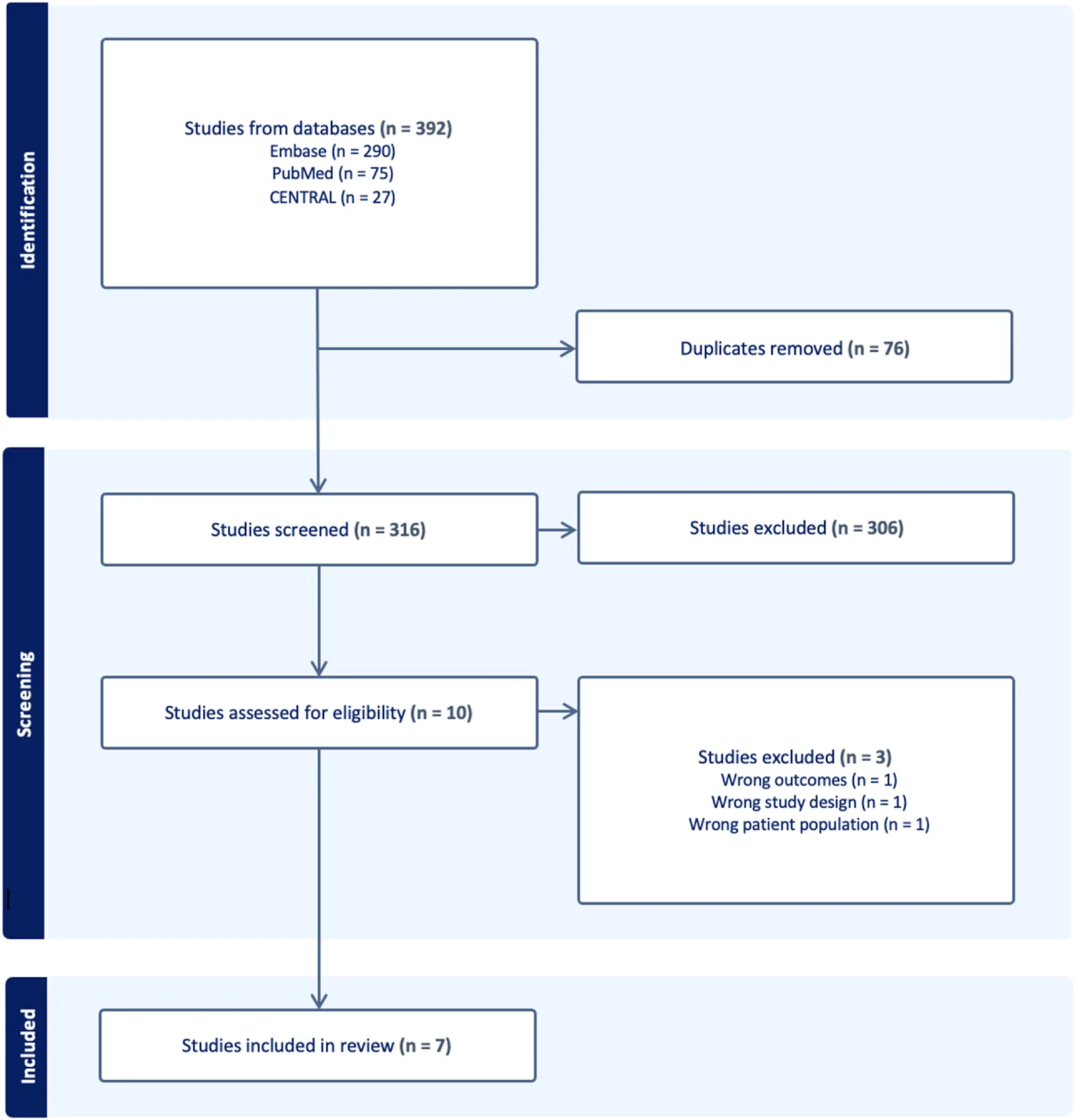
PRISMA flow diagram of study selection.

Two authors independently screened titles, abstracts, and full texts using Covidence systematic review software to identify relevant clinical studies. Studies were included if they involved patients undergoing surgical treatment for shoulder instability such as arthroscopy, Bankart repair, labral repair, Latarjet, remplissage, or capsulorrhaphy and examined BMI, obesity, or weight as a variable. Acceptable outcomes included recurrent instability events, revision surgery, patient-reported outcomes, and functional measures. Only primary research studies including randomized controlled trials, cohort studies, or case-control studies were considered. Exclusion criteria included studies involving rotator cuff repair, shoulder arthroplasty, joints other than the shoulder, case reports or case series with fewer than five patients, and secondary literature such as systematic reviews or meta-analyses. Discrepancies in study inclusion were resolved by a third independent reviewer. The initial search yielded 392 studies, including 290 from Embase, 75 from PubMed, and 28 from Cochrane. Seven studies met inclusion criteria and were included in the final review (Fig. 1). Study-specific outcomes were determined after inclusion.

Fig. 1 Caption: Studies identified through database are shown along with the screening inclusion and exclusion via the Covidence software. After removal of duplicates, titles and abstracts were screened followed by full-text review, resulting in inclusion of 7 studies.

Data extracted from each study included the journal and author, year of publication, level of evidence, sample size, patient sex and age, BMI, lifestyle and comorbidity status, type of instability surgery, relevant radiologic findings when available (such as Bankart lesion, glenoid bone loss, and Hill-Sachs lesion), duration of follow-up, and reported outcomes. Outcomes included functional measures such as Patient-Reported Outcomes Measurement Information System (PROMIS) scores, patient-reported outcomes including Rowe and American Shoulder and Elbow Surgeons (ASES) scores, recurrent instability or complications, and return to play.

## 3 Results

### 3.1 Characteristics of included studies and patients

Of the 392 identified studies, 7 studies (1.8%) met formal inclusion criteria. Of these studies, 5 were level III evidence and 2 were level IV evidence. All included studies were retrospective cohort studies except for 2, which were case series (Table 1). Randomized control trials assessing BMI and its impact on shoulder stabilization surgery were unavailable.

**Table 1:** Characteristics of the included studies (n = 7).

Level of Evidence	Column 1
Level I	0
Level II	0
Level III	5
Level IV	2
Publishing Journal	
The American Journal of Sports Medicine	1
JSES International	1
Arthroscopy: The Journal of Arthroscopic and Related Surgery	2
Orthopaedic Surgery	1
JAAOS Global Research & Reviews	1
Journal of Shoulder and Elbow Surgery	1

Table 1 Caption: The level of evidence as well as the publishing journal for each study analyzed in this review.

A total of 936 patients were pooled from the included studies. The mean age from these patients was 31.9 years and the mean BMI was 29.74. There were 630 male (67.3%) patients, 163 female (17.4%), and 143 (15.3%) for which no gender was specified in their respective study. The mean clinical follow-up time across all studies was 24.3 months (range, 10.3-53.8) (Table 2).

**Table 2:** Clinical and demographic characteristics of the included patients (n = 936).

Characteristics	Value
Total # of Patients	936
Sex	
Male	630
Female	163
Not Specified	143
Mean Age	31.96 ± 7.99
Mean BMI	29.74 ± 7.72
Mean Follow-Up (Months)	24.32 ± 15.09
Type of Surgical Procedure	
Bankart Repair	301
Bankart + Capsulorrhaphy	57
Autologous Scapular Spine Bone Graft	27
Latarjet	60

Table 2 Caption: The relevant demographic information of patients included across all studies along with the number of patients in each category.

Influence of BMI on Shoulder Instability Outcomes.

Of the included studies, one study explicitly evaluated BMI as the primary independent variable by stratifying patients into BMI-based cohorts and comparing surgical outcomes across these groups.^1^ The remaining six studies included BMI as a variable in their analysis but did not focus on it as the primary factor of interest.^14^, ^15^, ^16^, ^17^, ^18^, ^19^ Within these studies, there were no differences demonstrated in surgical complications, instability recurrence, revision rates, or patient-reported outcomes between groups (Table 3). Yang et al. demonstrated no significant correlation between BMI and graft resorption rate in a study evaluating autologous scapular spine bone graft for patients with recurrent anterior instability. Additionally, Papalia et al. found no significant difference in the percentage of glenoid bone loss, number of Hill-Sachs lesions, or patient-reported outcomes between various BMI cohorts. However, obese patients had less improvement in upper extremity function when compared to patients with normal BMI (18-25) as assessed by the PROMIS score.^13^ Regarding the Minimal Clinically Important Difference (MCID) for two patient-reported outcome measures, Park et al. found that elevated BMI was negatively correlated with the MCID for the Rowe score, but there was no association between BMI and the MCID for ASES scores.

**Table 3:** Summary of study findings on BMI and shoulder instability.

Study	Key BMI-Related Findings	BMI Effect on Instability/Outcomes	BMI Classification
**1. Graft Resorption (Scapular Spine Graft)**	No correlation with graft resorption	No significant effect (P = 0.825)	Continuous Variable
**2. Soft Tissue Arthroscopic Repair**	Longer surgical time and ↑ partial RC tears in obese	No effect on recurrence/revision (P > 0.05)	Cohorts (BMI<25, BMI≥25 and < 30, BMI≥30
**3. Nonoperative PSI Management**	Trend toward more surgery in BMI ≥35	Trend toward increased surgery (P = 0.10)	Continuous Variable
**4. Posterior Instability & Bone Lesions**	Higher BMI in BCL group (univariate only)	Not significant in multivariate analysis	Continuous Variable
**5. Return to Sport After Latarjet**	Higher BMI = lower RTS odds (OR = 0.615)	Significant negative predictor (P = 0.016)	Continuous Variable
**6. MCID Post-Arthroscopic Stabilization**	Higher BMI = lower MCID for Rowe score	Significant negative correlation (P = 0.01)	Continuous Variable
**7. GH Dislocations in BMI ≥ 40**	↑ Bankart lesions with higher BMI; no link to recurrence	No significant effect on recurrence/surgery	Continuous Variable

Table 3 Caption: Summary of key findings related to BMI and shoulder instability surgery from each of the included studies.

Increased BMI was associated with a trend toward increased surgical intervention for patients with BMI >35, with a hazard ratio of 2.32 (95% CI: 0.8-6.8). Woodmass et al. also showed that BMI >35 was predictive of late conversion to surgery in patients originally receiving non-operative treatment. The obese cohort (BMI >30) had greater surgical duration (99.8 ± 40.0 vs. 75.7 ± 28.5 min) and increased incidence of partial rotator cuff tears (60% vs. 27%, odds ratio: 3.2 [1.1, 9.2]). One study by Lansdown et al. reported patients with shoulder instability and associated bone and cartilage lesions (BCLs) had a significantly higher mean BMI compared to those without BCLs (28.1 ± 4.8 kg/m2 vs. 26.5 ± 4.8 kg/m2). However, multivariate logistic regression did not identify BMI as an independent risk factor for BCLs. Maheshwer et al. focused on outcomes after shoulder dislocation in individuals with morbid obesity, defined as BMI >40. Amongst morbidly obese patients, those with Bankart lesions and recurrent dislocations tended to have higher BMI. Additionally, Gowd et al. found that increased BMI was associated with decreased likelihood of returning to sport following the Latarjet procedure.

## 4 Discussion

The impact of elevated BMI on outcomes after surgery on weight-bearing joints is well established; however, its influence on outcomes after shoulder stabilization surgery has not been as thoroughly investigated.^3^^,^^4^ This systematic review synthesizes current evidence to better understand the implications of BMI on shoulder instability surgery. In contrast to findings from studies on lower-extremity joints, elevated BMI did not emerge as a statistically significant independent predictor of poorer postoperative outcomes, namely recurrent instability or revision following shoulder stabilization surgery.^20^ Furthermore, this differs from studies showing increased operative complications after reconstructive shoulder surgeries in patients with obesity.^6^^,^^21^ Nonetheless, several consistent and clinically relevant associations were identified between BMI and other aspects of shoulder instability and associated surgical treatment, particularly with severity of shoulder pathology and complexity of surgical repair.

Papalia et al. found no difference in recurrence, revision rates, or patient-reported outcomes based on BMI stratification. This contrasts directly with studies published on total knee arthroplasty (TKA), total hip arthroplasty (THA), and even total shoulder arthroplasty (TSA). A robust body of evidence suggests a positive relationship between elevated BMI and the need for surgical revision and lower patient-reported outcomes in both TKA and THA.^22^^,^^23^ Similar findings were demonstrated in a study investigating the effects of BMI on TSA.^21^ This difference in outcomes and revision rates is perhaps easier to understand between the shoulder instability and TKA and THA populations as joint replacements of the lower extremity are directly impacted by increased axial load. For example, a significant portion of the revisions in the study of TKA above were due to prosthesis loosening, a problem largely understood to be impacted by increased body weight.^23^ The differences between postoperative outcomes in shoulder instability and shoulder arthroplasty studies in the context of BMI may be more complex; however, mechanical failure, as commonly seen in TKA, also contributes to these variations.^21^ Thus, the lack of significant mechanical hardware in most stabilization surgeries could be a large factor in fewer documented postoperative issues and need for surgical revision.

Notably, two studies demonstrated significant relationships between BMI and functional outcomes following shoulder stabilization surgery. Higher BMI was a significant negative predictor of return to sport following Latarjet reconstruction, indicating that patients with higher BMI were less likely to return to preinjury levels of activity.^17^ Similarly, obese patients undergoing surgery for ligamentous knee injuries display decreased return to sport compared to non-obese patients.^24^, ^25^, ^26^ While the shoulder is not a weight-bearing joint, higher BMI negatively affecting return to sport after Latarjet reconstruction may reflect both biomechanical and physiologic factors. Increased body mass can place greater demands on stabilizing structures and may impair graft healing, while obesity-related inflammation and reduced conditioning may further limit functional recovery. Similar associations have been observed in lower-extremity injuries, suggesting that BMI represents a cross-joint factor influencing musculoskeletal outcomes. These results highlight the importance of considering BMI in preoperative counseling and rehabilitation planning. Furthermore, elevated BMI was associated with a lower MCID for the Rowe score after arthroscopic shoulder stabilization for anterior shoulder instability.^19^ Other studies investigating various orthopedic procedures from total joint arthroplasty to spine surgery have demonstrated similarly decreased MCID thresholds in patients with elevated BMI.^27^, ^28^, ^29^ This suggests that patients with elevated BMI may perceive meaningful clinical improvement with smaller objective gains. Studies have shown lower baseline functional scores amongst patients with elevated BMI which may contribute to relatively lower threshold for subjective improvement after surgery.^30^^,^^31^ Furthermore, patients with elevated BMI may have lower activity levels and functional demands after surgery which could decrease the threshold for patient satisfaction. While difficult to quantify, these findings suggest that BMI may influence not only outcomes but also patient perception of surgical success, a consideration that may help guide interpretation of outcome metrics and patient satisfaction.

Beyond functional recovery, several studies in our review also linked elevated BMI to specific injury patterns in shoulder instability. A higher prevalence of Bankart lesions in patients with BMI ≥40, greater incidence of partial rotator cuff tears in obese individuals undergoing Bankart repair, and bone and cartilage lesions in those with higher BMI was observed.^13^^,^^16^^,^^18^ Although one supporting study found no statistically significant link between obesity and rotator cuff tears, they noted increased obesity prevalence and metabolic disorders in individuals with rotator cuff pathology, suggesting a possible underlying relationship.^32^ Other studies have identified a significant, positive relationship between obesity and shoulder joint pathology. Various studies have found an increased prevalence of rotator cuff atrophy and tear amongst obese patients.^33^, ^34^, ^35^ The mechanism behind increased prevalence and severity of shoulder pathology amongst patients with elevated BMI is likely multifactorial. Biomechanical factors such as increased joint stress, altered loading patterns, and capsular laxity may contribute to the observed variations. Similar factors have been implicated in pathology in other joints. For example, obesity-related osteoarthritis progression in the hip due to increased contact forces and unfavorable biomechanics and significantly altered ligamentous laxity in the knee due to excess adiposity have both been well documented.^36^^,^^37^ These physiologic differences may help explain the higher incidence of specific soft tissue or bony injuries in patients with elevated BMI and shoulder instability.

Another consistent trend across studies was the association between elevated BMI and increased surgical complexity. Papalia et al. found significantly longer operative times in obese patients undergoing shoulder stabilization surgery. Similar findings have been reported with joint arthroplasty procedures.^13^^,^^38^ This increase in operative time is likely due to the added challenges of exposure, retraction, and positioning in patients with greater soft tissue mass. Excess adipose tissue can significantly reduce visualization in both open and arthroscopic procedures. Several studies have shown that increased body mass is also associated with greater blood loss and more frequent intraoperative complications which can necessitate additional interventions and extend the duration of surgery.^39^^,^^40^ The elevated difficulty of positioning, exposure, and intraoperative complications are all likely reasons that surgeries tend to be more complex and require more time for individuals with elevated BMI. Collectively, these findings underscore the need for careful perioperative planning and risk stratification in patients with elevated BMI, as surgical demands and complexity may be higher even in procedures involving non-weight-bearing joints.

This review is limited by the retrospective nature of all included studies and the relatively small number of papers addressing this topic. These limitations hinder our ability to make definitive conclusions regarding causality and underscore the need for larger studies with standardized outcome reporting and higher statistical power. Furthermore, the heterogeneous nature of outcomes in the papers prevented a larger meta-analysis of available data. Until such data are available, clinicians should use the current findings to guide individualized patient care and optimize expectations and surgical planning in patients with elevated BMI.

## 5 Conclusion

Across a range of settings, elevated BMI has not consistently emerged as a direct, independent predictor of recurrent instability or revision surgery following shoulder stabilization procedures. However, our systematic review confirms that higher BMI frequently influences important aspects of patient outcomes, most notably operative complexity, injury patterns, functional recovery, and subjective improvement thresholds. Obese patients often require longer surgical times and may present with increased rates of Bankart and partial rotator cuff lesions, as well as report lower odds of returning to sport. Additionally, elevated BMI appears to alter patient perceptions of improvement following surgery, reflected in lower Rowe MCID thresholds. Collectively, these findings suggest that while BMI may not directly drive surgical failure, it plays a meaningful role in shaping both the technical and rehabilitative experience of shoulder stabilization. These insights mirror broader trends observed in hip and knee arthroplasty, where obesity complicates procedural logistics and recovery expectations. As such, when evaluating and counseling patients with elevated BMI, clinicians should emphasize tailored perioperative planning, individualized goal-setting, and realistic discussions about functional recovery. Future research should prioritize prospective, adequately powered studies with longer follow-up periods and standardized outcome measures, including both objective and patient-reported data, to fully elucidate the nuanced impact of BMI on shoulder instability management.

## Compliance with ICMJE authorship criteria

All authors meet criteria.

## Guardian/Patient's consent

Not applicable. This study is a systematic review of previously published literature and did not involve direct patient contact or the use of identifiable patient data.

## Ethical statement

Not applicable. Ethical approval was not required for this study as it is a systematic review of published data and did not involve human participants or animals.

## CRediT author statement

Bryce C. Johnson: Formal analysis; Investigation; Project administration; Visualization; Writing – original draft; Writing – review & editing.

Erryk S. Katayama: Conceptualization; Data curation; Formal analysis; Investigation; Methodology; Writing – review & editing.

Benjamin L. Brej: Writing – original draft; Writing – review & editing.

Kavya B. Ajjarapou: Writing – original draft; Writing – review & editing.

Gregory L. Cvetanovich: Conceptualization; Supervision; Writing – review & editing.

Julie Y. Bishop: Conceptualization; Supervision; Writing – review & editing.

Ryan C. Rauck: Conceptualization; Supervision; Writing – review & editing.

## Funding statement

This research received no external funding.

## Conflicts of interest

None.
